# Transcriptional Basis for Differential Thermosensitivity of Seedlings of Various Tomato Genotypes

**DOI:** 10.3390/genes11060655

**Published:** 2020-06-16

**Authors:** Yangjie Hu, Sotirios Fragkostefanakis, Enrico Schleiff, Stefan Simm

**Affiliations:** 1 Department of Biosciences, Molecular Cell Biology of Plants, Goethe University, D-60438 Frankfurt am Main, Germany; huyangjie850909@gmail.com (Y.H.); fragkost@bio.uni-frankfurt.de (S.F.); 2 Buchmann Institute for Molecular Life Sciences, Goethe University, D-60438 Frankfurt am Main, Germany; 3 Frankfurt Institute of Advanced Studies (FIAS), D-60438 Frankfurt am Main, Germany; 4 Institute of Bioinformatics, University Medicine Greifswald, D-17475 Greifswald, Germany

**Keywords:** heat stress, thermotolerance, *Solanum lycopersicum* L., Massive Analysis of cDNA Ends (MACE), seedling, transcriptome, RNA-seq

## Abstract

Transcriptional reprograming after the exposure of plants to elevated temperatures is a hallmark of stress response which is required for the manifestation of thermotolerance. Central transcription factors regulate the stress survival and recovery mechanisms and many of the core responses controlled by these factors are well described. In turn, pathways and specific genes contributing to variations in the thermotolerance capacity even among closely related plant genotypes are not well defined. A seedling-based assay was developed to directly compare the growth and transcriptome response to heat stress in four tomato genotypes with contrasting thermotolerance. The conserved and the genotype-specific alterations of mRNA abundance in response to heat stress were monitored after exposure to three different temperatures. The transcripts of the majority of genes behave similarly in all genotypes, including the majority of heat stress transcription factors and heat shock proteins, but also genes involved in photosynthesis and mitochondrial ATP production. In turn, genes involved in hormone and RNA-based regulation, such as auxin- and ethylene-related genes, or transcription factors like HsfA6b, show a differential regulation that associates with the thermotolerance pattern. Our results provide an inventory of genes likely involved in core and genotype-dependent heat stress response mechanisms with putative role in thermotolerance in tomato seedlings.

## 1. Introduction

The exposure of plants to heat stress (HS), even for a short period, has a significant impact on growth and development [1,2]. HS, among other stresses, negatively affects germination, developmental transitions, sexual reproduction, vegetative growth, photosynthesis, and causes cell cycle arrest [3,4]. At the molecular level, high temperatures disturb protein homeostasis and alter cellular metabolism. The latter causes alterations at primary and secondary metabolites resulting in the production of osmolytes and reactive oxygen species (ROS) scavengers [5,6]. In addition, phytohormones such as abscisic acid (ABA), ethylene and salicylic acid are increased in response to HS, while auxin, cytokinin and gibberellins are reduced [7,8,9,10]. 

The ability of plants to withstand an acute HS incident is defined as basal thermotolerance [11]. Basal thermotolerance is dependent on pre-existing molecular pathways that are rapidly activated during HS to confer protection and eventually survival. The genetically predisposed limits of basal thermotolerance can be exceeded when an acclimation treatment is applied prior to an otherwise lethal stress [12]. The latter is assigned as acquired thermotolerance. 

In both, basal and acquired thermotolerance, survival and recovery from HS are dependent on the activation of defense pathways collectively called HS response (HSR). The HSR is mainly regulated by HS transcription factors (Hsfs) [13]. Hsfs, as well as other transcription factors, control hundreds of genes induced in response to HS, including genes coding for proteins involved in DNA and RNA regulation, protein synthesis, metabolism, signaling, photosynthesis and hormone metabolism [14,15,16,17,18]. A central hallmark of HSR, however, is the Hsf-dependent transcriptional activation of additional Hsfs and of molecular chaperones such as heat shock proteins (HSP) [19]. HSPs are involved in protein quality control by protecting proteins from misfolding and terminal aggregation. Thereby, they are key components for the maintenance of protein homeostasis under stress conditions [20].

Studies to define HS-related processes frequently make use of tomato (*Solanum lycopersicum* L.), which in addition to serving as model for HSR is an important crop [17,21,22]. On the one hand, the thermotolerance mechanisms in tomato have been examined at molecular, physiological and genetic levels and the results have fueled basic models describing the regulation of HSR in plants [23,24]. On the other hand, tomato is cultivated in areas around the globe that are or will be affected by global warming [25]. Therefore, the generation of genetic tools for the selection and/or development of a more thermotolerant germplasm are important.

It was recently shown that there is a natural variation in tomato thermotolerance. Wild tomato species have a high capacity to rapidly acclimate to temperature increases, while modern genotypes show increased short-term acquired thermotolerance when compared to the wild tomato [24]. However, the HS resilience of tomato genotypes to acute exposures to different temperatures and their correlation with transcriptome changes have not been well explored. Typically, thermotolerance is determined based on prolonged stress treatments in contrast to transcriptome analyses that are frequently investigated after short HS exposures [26]. Therefore, a direct link between phenotypic and transcriptome observations, which would allow the generation of an inventory of putative thermotolerance-related genes, is not established yet.

We have developed a thermotolerance assay for tomato which can be extended to other plant species or even to cross-species studies. It is based on hypocotyl elongation rate of tomato genotypes after a short stress treatment in a series of temperatures ranging from mild to severe HS. We describe the temperature-dependent behavior of the seedlings and compare these results with a hypocotyl transcriptome analysis on four genotypes with contrasting thermotolerance. Based on our findings we discuss global HSR patterns and candidate genes that are likely related to global or to genotype-specific thermotolerance.

## 2. Materials and Methods

### 2.1. Plant Growth and Treatment

*S. lycopersicum* seeds from four genotypes (Moneymaker, Red Setter, LA2661 (Nagcarlang) and LA1994) were surface sterilized by ethanol and sodium hypochlorite. The sterilized seeds were placed on wet paper towels in Petri dishes and allowed to germinate under constant 25 °C in the dark. Four-day-old seedlings with approximately 1 cm hypocotyl length were exposed for 1.5 h to the indicated temperatures (25 °C, 40 °C, 42.5 °C, 45 °C, 45.7 °C, 50 °C) and then allowed to recover for five days in the dark at 25 °C. Directly after the treatment and every 24 h, the seedlings were photographed and their hypocotyl length was determined using ImageJ [27]. The values obtained by the independent experiments were combined and analyzed by non-linear least square fit analysis with Sigma Plot (Systat Software GmbH, Erkrath, Germany) using Equation (1)

Growth = max/(1 + (x/T_50%_)^(-H)) (1)

With H being the Hill-slope and T_50%_ the temperature at which 50% of the growth reduction in response to the elevated temperature is observed. The T_50%_ is a measure of the thermotolerance. The Hill-slope (H) is a measure of the thermosensitivity, because this parameter is proportional to 1/log_10_(EC_90_/EC_10_) defining the temperature range in which a plant can respond to HS by modulation of the cellular state (eustress). Hence, a lower absolute value of the Hill coefficient represents a larger temperature range in which growth still can occur.

### 2.2. RNA Extraction and NGS Analysis

Seedlings of the four genotypes were grown as described above, exposed for 1.5 h to 25 °C, 39 °C or 45 °C and then returned to 25 °C for 1.5 h. Seedlings kept at 25 °C served as control. A temperature 39 °C was selected as it is just below the EC_90_ value observed for all genotypes and thus represents the state where all genotypes can tolerate HS. The transcriptome after a 45 °C treatment was analyzed, which is in the range of the T_50%_ of at least three genotypes and of the EC_10_ value for Red Setter. The total RNA was extracted from hypocotyls of at least six seedlings per replicate and condition directly after the HS application and after 1.5 h recovery using the EZNA Plant RNA kit (Omega Bio-Tek, Norcross, GA, USA) according to the manufacturer’s protocol. For the transcriptome analysis, equal amounts of total RNA from HS and HS-recovery samples were pooled. This way, information for transcriptome alterations occurring during and after HS were obtained simultaneously, as both phases involve metabolic changes important for thermotolerance. The cDNA was synthesized using the reverse transcriptase RevertAid (Thermo Fisher, Carlsbad, CA, USA) according to manufacturer’s instructions. The quality of total RNA was checked with Bioanalyzer, and Massive Analysis of cDNA Ends (MACE) libraries were prepared based on GenXPro (Frankfurt am Main, Germany). In MACE, each read corresponds to one transcript of the total RNA sample and the abundance of a transcript is proportional the abundance of the particular read. Further, using MACE, less reads are needed to quantify the abundance of a particular transcript when compared to standard mRNA-Seq of entire transcripts, where the quantification depends on the coverage of the gene as well.

Poly-adenylated RNA was extracted with a Dynabeads mRNA Purification kit (Life Technologies, Grand Island, NE, USA) and reverse transcribed with a SuperScript Double-Stranded cDNA Synthesis Kit (Life Technologies, Grand Island, NE, USA) using biotinylated poly(dT) primers. The fragmentation of cDNA was conducted with Bioruptor (Diagenode, Seraing, Belgium) which yielded an average size of 250 bp. The biotinylated cDNA ends were captured by M-270 Streptavidin Dynabeads (Life Technologies, Grand Island, NY, USA) and ligated with T4 DNA Ligase 1 (NEB, Ipswich, USA) to TrueQuant universal adapter (GenXPro, Frankfurt am Main, Germany). The PCR amplification was performed with KAPA HiFi Hot-Start Polymerase (KAPA Biosystems, Wilmington, NC, USA). The PCR products were purified by Agencourt AMPure XP beads (Beckman Coulter, Krefeld, Germany) and sequenced with HiSeq2000 (Illumina, San Diego, CA, USA).

### 2.3. Quantitative RT-PCR

Quantitative real-time PCR (qRT-PCR) was performed on a StepOnePlus cycler (Thermo Fisher Scientific, Waltham, USA). The reactions were performed in triplicates and each contained gene-specific oligonucleotides (Table S1), PerfeCTa^®^ SYBR^®^ Green FastMix Low ROX™ (Quanta BioSciencies, Gaithersburg, MD, USA) and the template, in a 10 μL total volume. The thermal cycling conditions were 95 °C/3 min followed by 95 °C/15 s, 60 °C/30 s, 72 °C/30 s for 40 cycles. Oligonucleotides (Table S1) were designed using PRIMER3 (www.genome.wi.mit.edu/cgi-bin/primer/primer3.cgi/). Data were analyzed by the ΔΔCt method [28] and presented as relative transcript levels using the EF1α (Solyc06g005060) gene as internal standard [29].

### 2.4. Read Alignment and Analysis of MACE Sequencing

Two independent MACE libraries (biological replicates) for each of the four different genotypes (LA1994, LA2661, Moneymaker and Red Setter) exposed to the three different temperatures (25 °C, 39 °C, 45 °C) were obtained. The analysis yielded between 5–10 million stranded reads of 100 bp for each replicate (Table S2; by GenXPro, Frankfurt am Main, Germany). A similar number of mapped reads and identified annotated genes was obtained for all genotypes, and only slight discrepancies could be observed for seedlings exposed to 45 °C with respect to the other temperatures (Table S2).

Each MACE library of reads was aligned to the genome of tomato (version ITAG2.4, cv. Heinz) provided by the Sol Genomics Network (SGN) [30]. First, a library quality control was performed via FastQC (https://www.bioinformatics.babraham.ac.uk/projects/fastqc/) to check for the duplication level and sequencing quality of the reads. For the alignment, NextGenMap (version 0.4.12; [31]) was used in single-end mode with default parameters with the following modifications: --kmer-skip 1 (number of seeds to skip; this modifies the sensitivity for finding all possible matching positions); --silent-clip (clipping the parts of reads not mapped without including this information in the output file (SAM); this simplifies the post-hoc handling of the SAM file); and --no-unal (unaligned reads are not shown in the SAM file; this saves space and simplifies the post-hoc analysis). The read alignments with a sum of insertions, deletions and mismatches greater than two were excluded.

For the quantification of transcript levels reads for all genes annotated in the Generic Feature Format version 3 (GFF3) file of tomato were counted with htseq-count of the High-Throughput Sequencing python framework (HTSeq; [32]). Transcript abundance was normalized to the library size for each sample yielding “transcripts per million mapped reads (TPM)”. The method is defined in Wagner et al. [33], but it was adapted for the MACE experiment by leaving out the transcript length scaling. This was performed to account for differences in the sequencing depths among the MACE libraries of individual samples (adapted from Conesa et al. [34]). Further normalization was not required because of the feature of MACE that one transcript is represented by only one read.

### 2.5. Principal Component Analysis (PCA)

The mean expression was calculated from the two biological replicates per temperature and genotype. The Principal Component Analysis (PCA) was performed using all MACE datasets (Section 3.2) or using the MACE datasets of all genotypes for one temperature (Section 3.3). For this analysis R with RStudio was used together with the libraries prcomp and factoextra. The contributions and values of the individuals (genes) and variables (samples) to single principal components (PC) as well as the variance explanation of PC was calculated (Tables S3–S5). The visualization of the PCA plots was performed by ggplot (R library) and Sigma Plot (Systat Software GmbH, Erkrath, Germany).

The protein coding genes contributing most to PC1 and PC2 were identified. The 25,478 genes that were found to be expressed considering the entire dataset were used as base. If each gene contributed equally to a given PC, the contribution would equal 0.003925%. Protein coding genes were considered as contributing the most to a given PC when the expression profile contributed at least tenfold more than the putative average of 0.003925% to this PC (PC1 or PC2). Therefore, each of the selected genes contributes with its transcript profile with at least 0.03925% to the PC (Tables S4 and S5).

### 2.6. Classification of the Genes

For the differential expression analysis of the cultivars under different temperatures, DESeq2 (version 1.26.0) was used (bioconductor.org/packages/release/bioc/html/DESeq2.html, [35]) in R (version 3.6.3, www.r-project.org) in the environment of RStudio (rstudio.com). For the pairwise comparison of transcript abundance between different genotypes at the same temperature, or between different temperatures of the same genotype, the Wald test (Table S6) was used. Genes were considered as differentially expressed when the adjusted *p*-value was lower than 0.01. The adjusted *p*-value was calculated based on the false discovery rate determined by the Benjamini–Hochberg procedure implemented in DESeq2.

The classification was based on the three ratios log_2_(TPM_39 °C_/TPM_25 °C_), log_2_(TPM_45 °C_/TPM_25 °C_) and log_2_(TPM_45 °C_/TPM_39 °C_). Differential expression with adjusted *p* < 0.01 was considered significant. Genes were subsequently classified into 15 classes: class 1–5 represented genes upregulated at 39 °C, class 6–10 represented genes downregulated at 39 °C, and 11–15 represented genes without any significant change between 39 °C and 25 °C. Further classifications considered the relation between 45 °C and 25 °C as well as between 45 °C and 39 °C, as described and discussed in Section 3.2.

Following, the similarity of classification of an individual gene in all genotypes was analyzed. (i) Genes were classified as level 1 in cases where the profile of transcript abundance was identical in all genotypes; this means that all log_2_ values and *p*-values yielded the same result (enhanced, reduced or not altered) (e.g., the expression of a gene was found in class 1 in all genotypes). (ii) Genes were classified as level 2 in cases where the log_2_(TPM_39 °C_/TPM_25 °C_) or the log_2_(TPM_45 °C_/TPM_25 °C_) yielded identical results (enhanced, reduced or not altered) for all genotypes to call for differential expression between the control and one HS condition (Section 3.2).

### 2.7. Functional Assignment and Voronoi Treemap Representation

The functional assignment was performed by the MapMan functional hierarchy ([36], Table S7) and a priori biological knowledge by manual curation. For some presentations, MapMan functional categories were merged, as indicated in the according figure legend.

The visualization via hierarchy-based Voronoi Treemaps was realized by an in-house application called VoronoiTreeraiser using the mean TPM values of the different genotypes and temperatures and functional annotation of MapMan as input. For the comparison of transcript abundance between all genotypes using the same interval, the relative abundance from the mean TPM value per gene for all genotypes in one treatment was calculated. First, for each gene in one genotype and in one treatment A(x)_(T(t)G(i))_ (mRNA abundance average for gene x for a given genotype i and temperature treatment t) was calculated based on the individual TPM values for the two replicates. Second, the relation of the expression of a given gene x at a given temperature T in the different genotypes was calculated by X(x)_T(t)G(i))_ = A(x)_(T(t)G(i))_/Σ_i_ A(x)_(T(t)G(i))_. By this method, the relative abundance per gene for all genotypes at one temperature was calculated similar to a z-score and used for visualization by a color-coded scale. These values were rescaled to a minimum (0, red) and maximum (1, blue) for all the Voronoi Treemaps. The statistical significance was analyzed by Kruskal–Wallis One-Way Analysis of Variance on ranks (*p* < 0.005) with normality, the Shapiro–Wilk normality test (*p* < 0.05) and the Tukey test for pairwise multiple comparison (*p* < 0.05) using Sigma Plot.

### 2.8. Hypothesis-Driven Gene Selection

For the hypothesis-driven gene selection, genes were selected according to the hypothesis formulated in Section 3.4, fulfilling the following criteria:

- Genes were selected in case a significant change in transcript abundance (*p* < 0.05) was observed for Red Setter at 25 °C, after 39 °C or after 45 °C HS treatment when compared to solely LA1994 and LA2661 at the according condition. Further, the change of transcript abundance between Red Setter and each of the other two genotypes, LA1994 and LA2661, had to be either positive or negative for both.
- Genes selected in (i) were further considered in case a difference of transcript abundance in Moneymaker (*p* < 0.05) at 25 °C or after 39 °C treatment was observed when compared to solely LA1994 and LA2661 at the according condition. Further, the change of transcript abundance between Moneymaker, LA1994 and LA2661 had to be positive or negative for both, and similar to the change found for Red Setter when compared to LA1994 and LA2661. These genes were selected as genes contributing to the difference between the tolerant and sensitive lines at 25 °C and 39 °C.
- Genes selected in (i) and fulfilling the following rule:
  log_2_(TPM_45 °C_ − TPM_25 °C_)_Red Setter_ > 0 and log_2_(TPM_45 °C_-TPM_25 °C_)_Red Setter_ − log_2_(TPM_45 °C_ − TPM_25 °C_)_Moneymaker_ > 1
  or
  log_2_(TPM_45 °C_ − TPM_25 °C_)_Red Setter_ < 0 and log_2_(TPM_45 °C_ − TPM_25 °C_)_Moneymaker_ − log_2_(TPM_45 °C_ − TPM_25 °C_)_Red Setter_ > 1
  were selected as genes contributing to the difference between tolerant and sensitive lines at 45 °C.

## 3. Results

### 3.1. Differences of Genotypes in Seedling Thermotolerance

**Figure 1:**
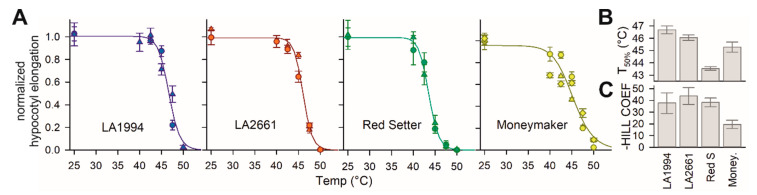
Temperature sensitivity of different genotypes. (**A**) The values for hypocotyl growth (cm day^−1^) were normalized to the growth of untreated seedlings and are presented as symbols for different biological replicas. The line represents the least square fit analysis of the data to Equation (1). Error bars are standard deviations for the different plants for one replica (*n* > 8). (**B**) The values of T_50%_ (EC50) for the four genotypes are shown. The standard error of the fit is shown as an error bar. (**C**) The values of the negative Hill-slope are shown. The standard error of the fit is shown as an error bar.

Four tomato genotypes were selected for the analysis. Two are described as thermotolerant (LA2661, alias “Nagcarlang”, and LA1994) by the Tomato Genetics Resource Center (TGRC) based on previous studies [37,38]. In addition, Moneymaker and Red Setter were included in the analysis. Moneymaker is a frequently used model in tomato HS research [14,24,39,40]. For Red Setter, a large TILLING population exists [41]. Thermotolerance was examined in seedlings and was defined based on the hypocotyl elongation capacity of seedlings after a short HS treatment ranging from 40 to 50 °C (Figure 1A). The inhibition of growth by HS for each genotype was analyzed by standard T_50%_ determination (Equation (1)). LA2661 and LA1994 have the highest T_50%_, followed by the intermediate value for Moneymaker, while Red Setter has the lowest T_50%_ (Figure 1B). Instead, based on the Hill co-efficient, Moneymaker has an increased eustress range, while the other three are similar (Figure 1C). The onset of growth inhibition under elevated temperatures for Moneymaker is as early as that found for Red Setter, while the 50% growth inhibition of Moneymaker is observed at the same temperatures as for the two more heat tolerant genotypes. Thus, LA2661 and LA1994 are more HS tolerant than Red Setter. Moneymaker shows a more gradual response range to heat that is intermediate when compared to the other three genotypes.

### 3.2. Common Transcriptome Responses of Tomato Genotypes to Elevated Temperatures

**Figure 2:**
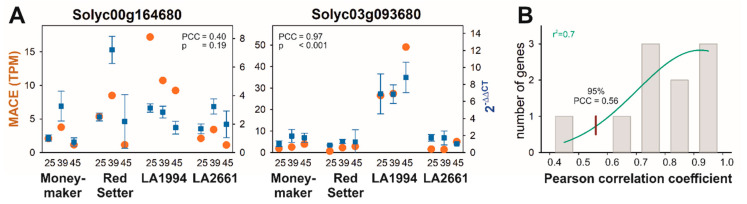
Correlation between Massive Analysis of cDNA Ends (MACE) and quantitative real-time PCR (qRT-PCR)-based transcript abundance quantification. (**A**) The transcript profiles for ten randomly selected genes were determined by MACE (TPM) or by qRT-PCR (2^−ΔΔCT^). The results for the lowest correlation (left) and the highest correlation are shown (right); other examples are in Figure S1. (**B**) The Pearson correlation coefficient (PCC) between MACE and the qRT-PCR profile was determined (grey bars) and its distribution analyzed by a Gaussian (green line). The red line indicates the PCC which occurs with 95% confidence. The r^2^ value for the least-square fit analysis is given.

A transcriptome analysis was performed on mRNA from hypocotyls of seedlings exposed to different temperatures to identify genes defining the differences in thermotolerance. Before the in-depth analysis of the transcriptome determined by MACE, the reliability of the MACE results was confirmed by quantitative RT-PCR on ten randomly selected genes (Figure 2A and Figure S1). The profile of the transcripts of gene with the lowest correlation between both experimental approaches differs in the transcript abundance in LA1994, while the global trend of the transcript abundance is conserved (Figure 2A, Solyc00g164680). The distribution of the Pearson correlation coefficient (PCC) between the expression profiles determined by MACE and by qRT-PCR was analyzed by a Gaussian distribution yielding a 95% confidence for a correlation of at least PCC = 0.56, which confirms the reliability of the MACE results (Figure 2B).

**Figure 3:**
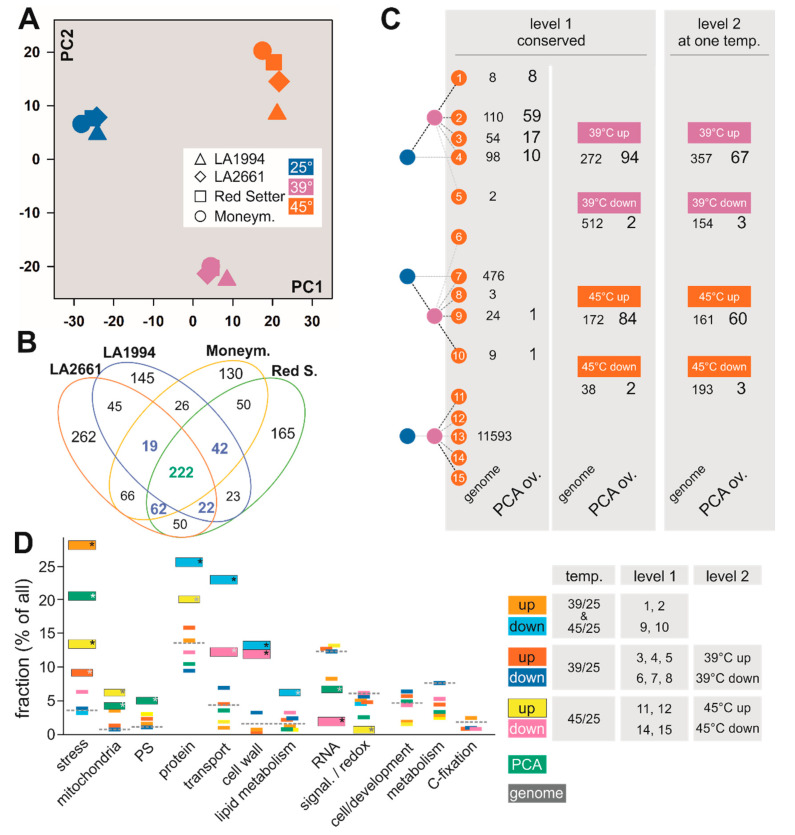
Common responses to different temperatures in the different genotypes. (**A**) Principle Component Analysis (PCA) for the transcript levels in the individual genotypes at different temperatures was performed using the average values of the two experiments. The distribution of PC1 and PC2 is shown. The symbol and color legend is indicated. (**B**) The genes contributing most to the PC1 and PC2 have been selected and the overlap between different genotypes is shown. (**C**) The expression profile of all genes (genome, small letters and numbers), and of the 222 genes found to define PC1 and PC2 in all genotypes (PCA ov., large letters and numbers) was classified as described in Section 2.6. Level 1 shows the genes with identical profile in all genotypes. For an overview, the number of genes up or downregulated at 39 °C or 49 °C in comparison to control is shown as well. Level 2 indicates genes with identical profile with respect to 39 °C/25 °C or 45 °C/25 °C, but not with respect to other comparisons. Note: (i) a gene in level 1 is not counted in level 2; (ii) for the accumulated information in level 1 and level 2, a gene can be counted twice. (**D**) All genes of the classes indicated on the right, selected by their contribution to PC1 and PC2 in all four genotypes (PCA overlap, green), or all genes in the genome (grey dashed line), were sorted into MapMan categories (Table S4). The percentage of genes in a given category was calculated in relation to the number of selected genes in a given class. The significance was determined by Fisher’s exact test (black/white asterisk: *p* < 0.01; grey asterisk: *p* < 0.05). C-fixation unifies categories 2–8, 25; cell and development: 31, 33, metabolism: 12–19, 22–24; signaling and redox: 20, 30 (Table S4). Miscellaneous/unknown, and DNA categories are not shown.

At a global level, all genotypes respond similarly to the different temperature treatments. In all genotypes, the calculated PC1 using the mean expression values for the two replicates (representing ~34% variance) separates the three different temperatures. PC2 (~23% of the variance) splits the response at 39 °C from the ground state at 25 °C and the state at 45 °C (Figure 3A and Figure S2; Table S3). 

The separation observed while using the using the mean expression values could be reproduced when individual experimental values were used for PCA (Figure S2). This justified the use of the mean values. The subsequent three PCs, irrespective of whether they were calculated with the mean or the individual values, discriminate the different genotypes (Figure S2). Thus, the main information for the stress response is included in PC1 and PC2.

The variance of the mRNA abundance pattern at different temperatures for each genotype was analyzed to identify the general response of tomato to increased temperatures at the transcriptome level. A PCA on the entire dataset was performed simultaneously to determine the linearly uncorrelated principal components (PC) describing the largest possible variance between the different temperature treatments in one cultivar. To determine the common response mechanisms for genotypes and/or temperatures, the mean TPM values were considered and genes that contribute most to PC1 and PC2 were selected (see Materials and Methods; Figure S2; Table S4). A total of 222 out of the 1329 different genes of PC1 and PC2 that have the highest contribution to PC1- and PC2-based separation (Section 2.5; Figure 3A) were identified in all four genotypes (Figure 3B green bold number). An additional set of 145 genes contribute to PC1 and/or PC2 in three genotypes (Figure 3B, blue bold numbers).

All genes were classified based on their differential expression at the three different temperatures based on values calculated with DESeq2 (Section 2.6, Figure 3C genome). The classification of all genes was compared with the one of the 222 highly contributing genes from PC1 and PC2 (Figure 3C PCA overlap). Of all genes, 62% (12,377) were classified identically in all four genotypes (Figure 3C, level 1, genome). Especially classes indicating an enhanced expression at 39 °C and 45 °C compared to 25 °C (classes 1, 2, 3), a downregulated expression at 39 °C only (class 7) or no alteration in expression (class 13) are highly populated. Of all genes with identical profiles in all genotypes, 784 genes are differentially expressed between 39 °C and 25 °C (higher: 512; lower: 27, level 1 right column, pink). For the differential expression between 25 °C and 45 °C, 172 genes were upregulated and 38 downregulated in all four genotypes (level 1 right column, orange). Considering the genes that are commonly upregulated or downregulated at 39 °C, although the general expression profile is not identical in all four genotypes, 357 (up) and 154 (down) genes were identified in addition (level 2). Thus, the transcripts of 629 genes are commonly upregulated and of 666 genes commonly downregulated in all genotypes at 39 °C. In total, 161 or 193 genes were upregulated or downregulated at 45 °C, respectively, in addition to the genes that show an identical profile (level 2). Hence, the transcript abundance of 333 genes has commonly higher and of 231 genes is commonly lower abundance at 45 °C.

The 222 genes found as highly contributing to the separation by PCA mostly represent upregulated genes (Figure 3C, class 1, 2, 3 of level 1, upregulation at 39 °C and 45 °C in levels 1 and 2). In total, at 39 °C the transcript abundance of 161 genes and at 45 °C the transcript abundance of 144 genes were upregulated. A manual inspection of the distribution of the changes of transcript abundance for genes contributing the most to PC1 and PC2 revealed that the genes with the largest alterations among the different treatments were selected (Figure S3). This suggests that the PCA analysis was efficient in discriminating differentially regulated from non-regulated genes, and that the PCA-based approach selected genes showing the strongest alterations in transcript abundance.

The abundance of the transcripts of genes coding for proteins assigned to different functional categories based on MapMan [36] was determined. To reduce the complexity, the classes representing genes upregulated or downregulated at 39 °C and 45 °C, as well as upregulated or downregulated at 39 °C or 45 °C when compared to 25 °C were merged (Figure 3D, legend). The transcript abundance of genes in different categories was compared to the abundance of the category in the entire genome (Figure 3D, dashed line). In addition, the 222 genes selected by the PCA-based method were categorized as well (Figure 3D, green).

Genes of the category “stress” response were significantly enriched among all genes with enhanced transcript abundance (orange, red, yellow) and in the pool of genes selected by PCA (green). In contrast, genes of the categories “signaling” and “redox regulation” show a significantly lower abundance in the set of genes with enhanced transcript level at 45 °C when compared to 25 °C (yellow). The genes of categories “photosynthesis” and “mitochondrial energy production” are significantly enriched among genes selected by PCA (green), and the latter also in the set of genes with higher transcript abundance at 45 °C (yellow).

Genes of the categories “transport” and “cell wall” are enriched among the genes with reduced transcript level at 39 °C and 45 °C (Figure 3D, blue) and at 45 °C only (magenta). Moreover, genes of the categories “protein” and “lipid metabolism” are enriched in the set of genes with reduced transcript abundance at 39 °C and 45 °C (Figure 3D, blue), while genes of the category “protein” are enriched among genes with enhanced transcript level at 45 °C (yellow). Worth mentioning, the category “RNA” is significantly less abundant in the pool of genes selected by PCA or genes with reduced transcript levels at 45 °C. No significant enrichment of genes in the other categories, such as development, metabolism or C-fixation, was found.

### 3.3. Variation in the Transcriptome Profile of Tomato Genotypes at Different Temperatures

**Figure 4:**
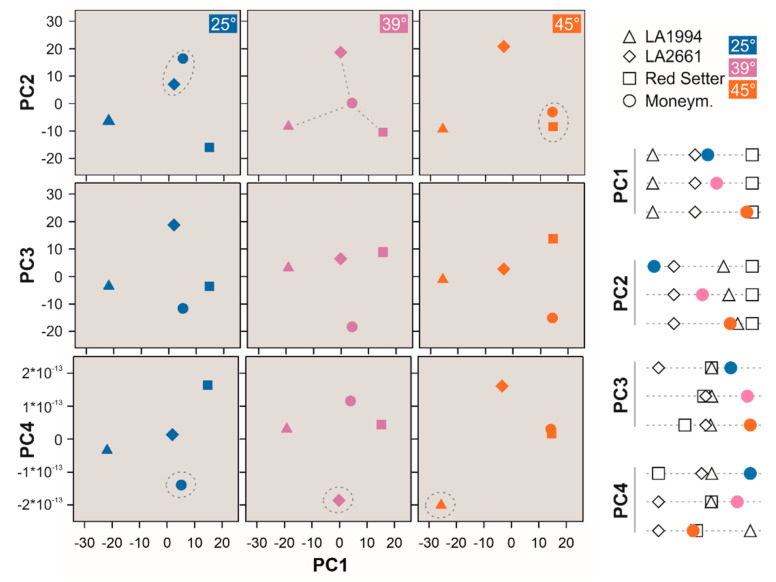
Principal Component (PC) analysis for different genotypes at a given temperature. The PC1 and PC2 (top), PC3 (middle) or PC4 (bottom) of the distribution of the transcript levels (mean values) in the different genotypes was calculated and is shown for each temperature. The symbol and color legend are indicated on the right. The circle and the lines are drawn for the visualization of the relations. On the right, the distribution of the genotype in each PC at each temperature is illustrated. The distribution of the individual experiments is shown in Figure S4.

To determine globally the genotype-dependent differences of seedlings exposed to normal or elevated temperatures the expression profiles of the different genotypes for each individual temperature were subjected to a second PCA (Figure 4, Figure S4, Table S5). The PCA-based approach was chosen because methods like hierarchical clustering rely on “human pattern recognition” [42]. More importantly, PCA has been introduced to identify important gene sets in multi-dimensional datasets [43]. It needs to be noted that PCA is limited when the size of information is low in comparison to the samples [44], which appears not to be the case here, with about 30,000 genes and 24 conditions (3 conditions, 4 genotypes, 2 biological replicates); or in case of limited information in comparison to experimental fluctuations [44]. The number of conditions was further reduced by using the mean of two experimental conditions, which in addition reduced the variation of information not related to the tested hypothesis.

PC1 (43% of the variation) discriminates LA1994, LA2661 and Red Setter at each of the three temperatures (Figure 4, Table S5). PC2 (31% of the variation) separates the expression profile of LA2661 from that of LA1994 (Figure 4). Red Setter is separated at 25 °C, but its expression profile becomes more closely related to that of LA1994 at higher temperatures (Figure 4). Thus, LA2661 seems to be more distinct in its expression profile from LA1994 than Red Setter, although LA2661 and LA1994 show a comparable thermotolerance (Figure 1). PC3 (25% of the variation) unifies the expression profile of LA2661 and LA1994 (Figure 4). Red Setter is clustered at 25 °C with these two genotypes but becomes distinct at 45 °C (Figure 4). PC4 (<1% of the variation) shows an increasing discrimination between LA2661 and LA1994 with increasing temperatures, while Red Setter is distinct at 25 °C, comparable to LA1994 at 39 °C and but also distinct from the other two genotypes at 45 °C (Figure 4).

The expression profile of Moneymaker shows a temperature-dependent transition considering PC1 and PC2 (Figure 4). At 25 °C the expression profile of Moneymaker is similar to that of LA2661 (Figure 4, blue circle). After the 39 °C treatment, the expression profile of Moneymaker is intermediate between LA2661 and Red Setter (PC1) or LA2661 and LA1994/Red Setter (PC2; Figure 4, purple circle). The exposure at 45 °C yields an expression profile of Moneymaker similar to Red Setter (PC1, Figure 4, orange circle) or Red Setter/LA1994 (PC2, Figure 4). PC3 and PC4 separate Moneymaker from the other genotypes based on their expression profiles, with the exception of PC4 at 45 °C, were the expression profile is comparable to that of Red Setter (Figure 4). In summary, especially the order obtained for PC1 is comparable to the thermotolerance profile, as LA2661 and LA1994 are distinct from Red Setter, and Moneymaker makes a transition from the more resistant to the more sensitive genotype with increasing temperatures.

**Figure 5:**
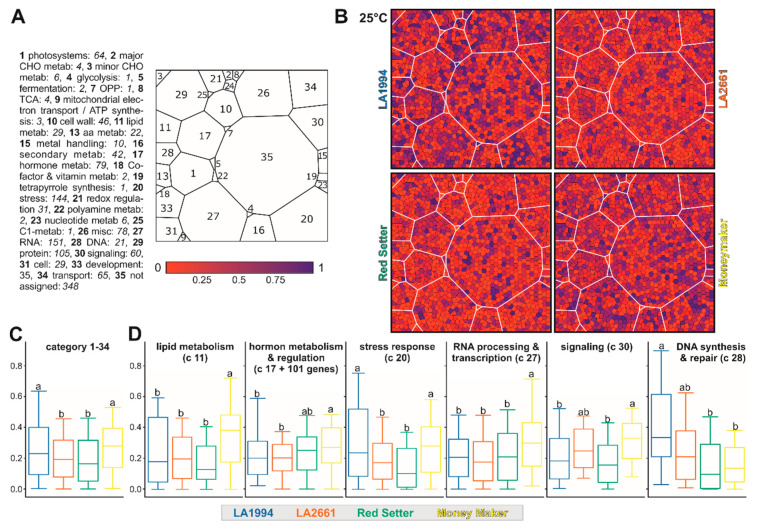
The genes contributing to PC1 and PC2 at 25 °C. (**A**) The scheme of transcript-level-based Voronoi Treemap representation according to categories is shown for orientation. On the right, the definition of the categories according to MapMan is given. The number of genes per category is indicated in italics. At the bottom, the color bar used in (**B**) is presented. The coloring was selected as such that red indicates a lower expression than expected by equal expression in all genotypes (which would equal 0.25), and violet and blue a higher expression. (**B**) The Voronoi Treemap of the transcript level for the indicated genotypes according to the scheme in (**A**) is shown. (**C**) The box plot representation of the distribution of the normalized transcript abundance values used for Voronoi Treemap representation is show for all genes (except not assigned). Statistical significance (*p* < 0.05) of the difference was tested by One-Way ANOVA (Tukey). (**D**) Box plot representation of the distribution of the normalized transcript abundance values used for the Voronoi Treemap in (**B**) is show for all genes of the indicated MapMan categories. For hormones, additional genes were selected as described in the text. Statistical analysis was performed as in (**C**). The color code for boxes in (**C**,**D**) accords to the name coloring in the legend.

To examine whether particular pathways contribute to the observed genotype-specific transcriptome variations, the 1393 genes contributing the most to PC1 and PC2 (methods) were assigned to MapMan functional categories [36]. To visualize differences among the different genotypes a Voronoi Treemap structure was created as described in Section 2.7 (Tables S5 and S8). The transcripts of most genes contributing to PC1 and PC2 at 25 °C are more abundant in LA1994 and Moneymaker compared to the other two genotypes (Figure 5A–C). This holds especially true for genes involved in stress response (Figure 5B,D). The transcript abundance of genes involved in lipid metabolism, hormone-based regulation, RNA-based regulation and signaling is higher in Moneymaker when compared to the other three genotypes, while the transcript levels of selected genes involved in DNA-based regulation are significantly higher in LA1994 (Figure 5B,D).

**Figure 6:**
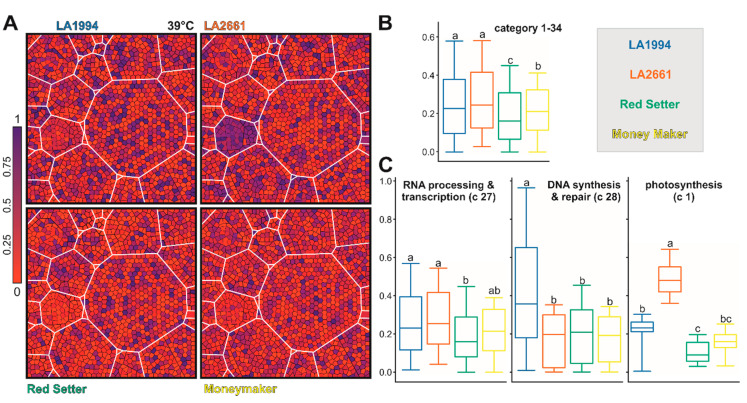
The genes contributing to PC1 and PC2 after 39 °C HS application. (**A**) The Voronoi Treemap representation of the transcript level for the indicated genotypes according to the scheme shown in Figure 5A is shown. The coloring (shown on the left) indicates a lower (red) or higher transcript abundance (violet and blue) than expected for an equal transcript abundance in all genotypes (= 0.25). (**B**,**C**) Box plot representations of the distribution of the normalized transcript abundance values used for the Voronoi Treemap in (**A)** for all genes (except not assigned; **B**) or for all genes of the indicated MapMan categories (**C**) is shown. Statistical significance (*p* < 0.05) of the difference was tested by One-Way ANOVA (Tukey test). The color code for the boxes accords to the name coloring of the legend.

After the 39 °C treatment, the transcript levels of the selected genes are generally highest in LA1994 and LA2661 (Figure 6A,B). The largest difference in transcript abundance is seen for genes involved in photosynthesis (blue in LA2661, Figure 6A category 1), which show higher expression in LA2661 (Figure 6C). Moreover, transcripts of genes involved in DNA-based regulation are still the most abundant in LA1994, while the transcripts of genes involved in RNA-based regulation show the lowest abundance in Red Setter (Figure 6C). 

**Figure 7:**
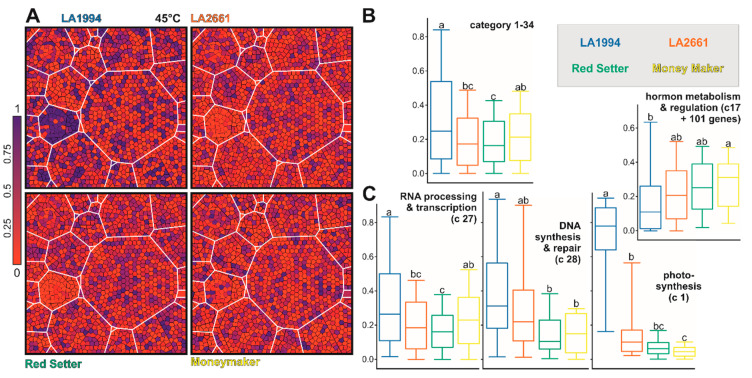
The genes contributing to PC1 and PC2 after 45 °C heat stress (HS) application. (**A**) The Voronoi Treemap as in Figure 5 of the transcript level for the indicated genotypes is shown. The coloring (shown on the left) indicates a lower (red) or higher transcript abundance (violet and blue) than expected for an equal transcript abundance in all genotypes (= 0.25). (**B**,**C**) Box plot representations of the distribution of the normalized transcript abundance values used in (**A**) for all genes (except not assigned; **B**) or for all genes of the indicated MapMan categories (**C**) is shown. Statistical significance (*p* < 0.05) of the difference was tested by One-Way ANOVA (Tukey). The color of the boxes accords to the color of the name in the legend.

At 45 °C, the transcript level of the selected genes is globally reduced in LA2661 and Red Setter when compared to LA1994 (especially photosystem, blue in LA1994, Figure 7A,B). Further, in LA1994 an overall higher abundance of the transcripts of genes involved in photosynthesis, RNA- and DNA-based regulation, cell function and transport was observed (Figure 7C, Figure S5; Tables S9–S11). In turn, the transcripts of genes involved in hormone-based regulation show the lowest levels in LA1994 at this temperature (Figure 7C).

Thus, a different transcript abundance of the PC1 and PC2 defining genes was observed in the different genotypes at 25 °C. Moreover, some of the categories like RNA-, DNA- or hormone-based regulation or photosynthesis unify genes that show differences even after exposure to higher temperatures. For instance, while the global transcript abundance pattern of the 61 photosynthetic genes contributing to PC1 and PC2 is not different between genotypes at 25 °C (Figure 5 and Figure S5), at 39 °C the transcript levels are more abundant in LA2661 and at 45 °C in LA1994 (Figure 6; Table S11). In LA1994 especially, transcripts of the genes coding for LHCPs are higher abundant levels at 45 °C.

Further, the global abundance of the transcripts of the 151 genes involved in RNA-based regulation at 25 °C is highest in Moneymaker, and after 45 °C treatment highest in LA1994 (Figure 5; Figure 7). The thermosensitive Red Setter shows a lower global abundance of the transcripts of these genes than in LA1994 after incubation at 39 °C or 45 °C, than found for LA2661 after 39 °C treatment, or than observed for Moneymaker after 45 °C stress (Figure 6 and Figure 7).

### 3.4. Specific Transcripts Involved in Cultivar-Specific Heat Stress Responses

**Figure 8:**
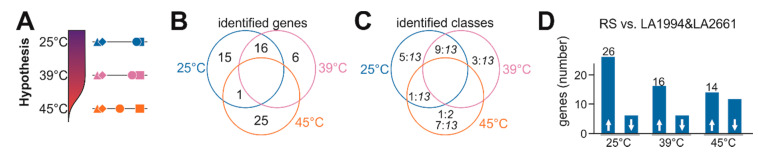
Analysis of genes differential regulated between HS sensitive and tolerant genotypes. (**A**) The hypothesis formulated on the basis of the physiological behavior is shown. (**B**) The number of genes selected by the described method (Section 2.8) and the overlap between the individual selections is shown. (**C**) The number of genes in specific class (number:class, as in Figure 3) is shown for the distribution presented in (**B**). (**D**) The number of genes with higher (first bar) or lower (second bar) transcript abundance at 25 °C (bar 1 and 2), 39 °C (bar 3 and 4) and 45 °C (bar 5 and 6) in Red Setter in comparison to LA1994 and LA2661 is presented.

The general response of the genotypes to HS at transcript level is rather comparable (Figure 3), while a genotype-dependent physiological response (Figure 1) reflected at different global expression patterns exist as well (Figure 4, Figure 5, Figure 6 and Figure 7). The analysis of the genotype specificity at the different temperatures yielded a discrimination of LA1994 and LA2661, both being more HS resistant than Moneymaker (intermediate) and Red Setter (thermosensitive; Figure 1). So far, the global response profile was analyzed (Figure 3, Figure 4, Figure 5, Figure 6 and Figure 7). To identify specific genes contributing to the contrasting physiological behavior, a hypothesis-driven approach was performed. This hypothesis considers that Moneymaker and Red Setter behave similar at normal and elevated temperature, but that Moneymaker shows a higher resistance to high temperatures than Red Setter (Figure 8A). Consequently, genes that are either expressed at significantly higher or lower levels as concluded from the observed transcript abundance in Moneymaker and Red Setter when compared to both LA1994 and LA2661 at 25 °C or 39 °C were selected. Further, genes with a higher or lower transcript abundance in Red Setter when compared to all other genotypes at 45 °C were collected as well.

This approach yielded 32 genes fulfilling the criteria at 25 °C, 22 at 39 °C and 26 at 45 °C (Figure 8B; Table S12). Interestingly, 16 genes are differentially regulated in Red Setter and Moneymaker at 25 °C and 39 °C, indicating that differences in gene expression at 39 °C exist already under non-stress conditions. In contrast, only one common gene between 25 °C and 45 °C exists, while no gene for 39 °C and 45 °C or all temperatures was identified. The identified genes belong to class 13 or do not show the same profile in all genotypes, with only one exception (Figure 8C). The genes selected as differentially expressed at 25 and 39 °C are mainly expressed at lower levels in the thermotolerant LA2661 and LA1994 lines indicating a possible negative relation to thermotolerance (Figure 8D). It appears that pre-existing transcriptome differences in non-stressed seedlings contribute to the physiological HSR. This can be concluded as many genes belong to class 13 and are thus not altered by strong up or downregulation in a specific cultivar.

We also found 14 genes with higher and 12 with lower transcript abundance in Red Setter at 45 °C when compared to the other three genotypes (Figure 8D). Here, the upregulated genes might contribute to the strong growth inhibition of the thermosensitive Red Setter under this temperature, while genes with reduced expression compared to the other genotypes might be involved in HS resistance. Specific genes are discussed below.

## 4. Discussion

### 4.1. The Global Response of Tomato Seedlings to Elevated Temperatures and Its Variability

Exposure to elevated temperatures is known to alter the plant transcriptome landscape [45]. We analyzed the transcriptome responses of four tomato genotypes with distinct thermotolerance capacities based on the hypocotyl elongation of seedlings after a short HS treatment. Despite significant variations in thermotolerance among the genotypes (Figure 1), common alterations in the HSR at the transcriptome level exist (Figure 3). There is a large overlap of genes contributing the most to the temperature-dependent differences in the transcriptome profiles. In response to 39 °C, 1295 genes are commonly altered in their transcript abundance in all genotypes, where most of them even show the same general profile in all genotypes as they belong to one of the classes 1–15. Exposure of plants to 45 °C yielded a set of 564 genes with either commonly enhanced or reduced transcript levels, but only one third of them showed an identical profile in all genotypes. Additionally, a large number of genes with reduced transcript abundance specifically at 39 °C were observed.

**Figure 9:**
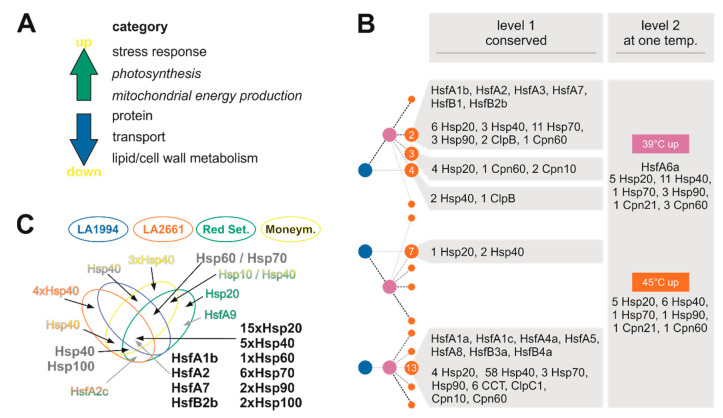
Hallmarks of the common response of tomato in response to elevated temperatures. (**A**) The categories enriched in upregulated or downregulated genes are shown. (**B**) The distribution of gene coding for heat shock proteins or the heat shock transcription factors in the different categories in Figure 3C is shown. (**C**) The distribution of gene coding for heat shock proteins or the heat shock transcription factors in the Venn diagram presented in Figure 3B is shown. Genes found in three species are shown in dark grey, genes shown in all species are shown in black, the others are color coded according to their occurrence.

There is an enrichment of genes involved in protein homeostasis, transport across membranes, and lipid or cell wall metabolism among the downregulated genes (Figure 9A). Moreover, genes assigned to the categories “photosynthesis”, “mitochondrial energy production” and “stress response” are enriched in the set of genes strongly altered in their transcript abundance (mostly upregulated in response to HS) identified by PCA-based approach (Figure 9A). An enrichment of genes of the category “mitochondrial energy production” is also found among genes with upregulated transcript levels at 45 °C. Thermotolerance is dependent on the activation of protective mechanisms during the stress period, which allow recovery upon return to physiological conditions. Recovery from stress is an energy-demanding process because mRNA transcription as well as the production of protective metabolites are involved [1]. Therefore, the high abundance of the transcripts of photosynthetic genes or genes involved in mitochondrial electron transport might be beneficial for stress resilience and the re-establishment of cellular growth. In the same direction, the downregulation of genes related to protein, lipid and cell wall synthesis (Figure 9A) might in part contribute to the reduction of energy demand. The latter is consistent with the growth reduction after HS.

Genes with generally upregulated transcript levels also often belong to the category “stress response”, which includes the core genes for HSR, namely HSF and HSPs (Figure 3; [39]). Out of the 196 genes of the tomato Hsp families (100, 90, 70, 60 40 and 20; [29], 63 are found to be differentially regulated (Figure 9B) and 47 have been identified by the PCA-based approach (Figure 9C). Many of the HSP family members are regulated in a similar manner and thus contribute to the common profile of HSR (Figure 9B,C). Remarkably, Hsp40-coding genes appear to have in part genotype-specific expression patterns. On the one hand, 58 Hsp40 genes are not differentially regulated in response to HS (Figure 9B, class 13). In turn, only seven genes are found in the same class, while 17 genes are commonly upregulated at 39 °C or 45 °C, but not at both temperatures (Figure 9B, level 1 vs. level 2). On the other hand, five Hsp40-coding genes define the PCs for all genotypes, while 10 define the PC for only a subset of genotypes (Figure 9C). Hsp40 proteins regulate the functionality and specificity of Hsp70 proteins [46] and thus, differences in expression of these genes might contribute to the genotype-dependent variation in thermotolerance. This is consistent with the observation that Hsp70-coding genes are by large commonly regulated in all genotypes (Figure 9B,C). Further, it is tempting to speculate that Hsp40s with opposing regulation (e.g., class 4 and 7) might have a counterbalanced function (Figure 9B).

The transcript level of seven of the 24 Hsfs [19] are found to be upregulated. The central regulators annotated as HsfA1b, HsfA2, HsfA3, HsfA7, HsfB1 and HsfB2b belong to the same class and show an enhanced transcript level at 39 °C and 45 °C when compared to the control condition in all genotypes (Figure 9B). The transcript of Hsf6a is induced only at 39 °C (Figure 9B). This Hsf might represent a link to hormone-based response because in *Arabidopsis thaliana* L. HsfA6a regulates ABA-related gene networks [47]. Four of the mentioned Hsfs are among the PCA-based selected genes in all genotypes and are thus among the most strongly regulated genes. The induction of HsfA2 and HsfB1 confirms the current model considering these are direct targets of the constitutively expressed HsfA1a, the master regulator of the tomato HSR (Figure 9B; [23,40]). For HsfB2b, a function in the regulation of hypocotyl growth at enhanced temperatures was described in *A. thaliana* [48], which is consistent with the upregulation of tomato HsfB2b found here.

PC3–PC5 describe genotype variations in general. This is not unexpected, because genotype-specific gene expression profiles with respect to following developmental stages can also occur, although the developmental stage examined here is comparable and all genotypes had similar length prior to the stress treatment.

### 4.2. Genotype-Specific Transcriptome Responses to Elevated Temperatures

Some genotype-specific features exist in addition to the common transcriptome response of all genotypes. Firstly, Hsp40s might in part be expressed in a genotype-specific manner (Figure 9). Secondly, the transcript of HsfA6a is enhanced in all genotypes at 39 °C but is differentially abundant at 45 °C, as judged from its assignment to level 2 (Figure 3). Thirdly, HsfA9 is only found in Red Setter to define the PCs (Figure 3; Figure 9C). Consistent with this observation, the analysis of the temperature-dependent recovery of hypocotyl growth yielded three distinct groups (Figure 1): LA1994 and LA2661 are the most heat tolerant, with the highest T_50%_ value and a rather steep transition from optimal to drastically reduced growth; Red Setter is the most heat sensitive based on the same parameters; Moneymaker shows an intermediate behavior.

The transcriptome profiles in part reflect the difference in the thermotolerance. The temperature-dependent transcriptome comparison among the genotypes (Figure 4) shows a clear distinction between LA1991, LA2661 and Red Setter considering the variance explained by the linearly uncorrelated PC1 and PC2. The intermediate behavior of Moneymaker becomes visible by the temperature-based analysis. Interestingly, the general profile of genes contributing the most to the variance at 39 °C or 45 °C reflect the thermotolerance descending order, meaning LA1994 and LA2661 as thermotolerant, Moneymaker as intermediate and Red Setter as sensitive ( Figure 6; Figure 7).

One category that almost reflects this regime, especially at 25 °C and 45 °C, includes genes related to hormone-based regulation. Several hormones play an important role in HSR and thermotolerance [3]. In particular, the transcript levels of genes involved in auxin (15 genes), ethylene (22 genes) and ABA-based regulation (38 genes) follow the thermotolerance regime, the latter only at 45 °C (Figure S6). Here, the global transcript abundance of genes involved in auxin-based regulation is generally higher in the thermosensitive genotype, while the transcript abundance of genes involved in ABA or ethylene-related processes is globally lower upon HS in Red Setter when compared to the other genotypes. Consistent with this observation, genes involved in hormone signaling belong to the genes that contribute significantly to the thermotolerance in positive or negative manner (Figure 8). For example, genes that are positively related to thermotolerance at more severe HS conditions (45 °C) include ABA2 (Solyc04g071960), an alcohol dehydrogenase that catalyzes the conversion of xanthoxin to abscisic aldehyde [49]. ABA has a positive role in thermotolerance and ABA2 mutant in *A. thaliana* is more thermosensitive than the wild type plants [50]. Therefore, a variation in the expression of ABA2 in tomato genotypes could serve as a possible marker for HS resilience. Similarly, the ethylene responsive factor 13/Jasmonate-responsive factor (ERF13/JER6; Solyc05g050790) is also expressed at higher levels in LA1994, LA2661 and Moneymaker at 45 °C, suggesting a positive role in thermotolerance. Members of the APETALA2/Ethylene Responsive Factor transcription factors act as mediators of stress responses [51]. In turn, Solyc03g093350, a protein likely involved in splicing and ethylene-dependent transcription, shows higher transcript levels in Red Setter and Moneymaker at 25 °C and 39 °C when compared to the thermotolerant genotypes (Table S12).

In addition, genes involved in RNA-based regulation and photosynthesis show a transcription profile that is comparable to the thermotolerance regime after 39 °C or 45 °C treatment. Remarkably, the photosynthetic genes most strongly contribute to the transcriptome variance in response to HS in hypocotyls (Figure 6; Figure 7). This is, however, reflected by a general change of the transcription of the genes rather than by a high abundance of particular genes, as only Solyc04g010190 coding a putative chloroplast localized chlorophyll binding protein is expressed at higher levels in the thermosensitive genotypes at 25 °C (Table S12).

Among the genes of the category “RNA”-based regulation most significantly contributing to the thermotolerance variation (Figure 8), one gene for the discrimination at 25 °C (Solyc05g006650) and six genes for the discrimination at 45 °C were detected. Four were lower expressed in Red Setter than in the other genotypes (Solyc04g018080, Solyc05g050790, Solyc05g013970, Solyc11g030380), and two were higher expressed in Red Setter (Solyc06g035940, Solyc04g056510). Solyc05g006650 is annotated as a bHLH family protein ZCW32 and is related to AT1G59640.1/AtbHLH031, which in *A. thaliana* regulates growth in an auxin-dependent manner [52]. Solyc04g018080 codes for an APFI-like transcription factor, Solyc05g013970 is annotated as RNA-binding protein 39, and Solyc11g030380 codes for a MADS box interactor-like factor (Solyc11g030380), all of yet unknown function. Solyc05g050790 is involved in the regulation of the ethylene response as discussed above. Hence, these four genes of the category “RNA”-based regulation might contribute to thermotolerance. The other two proteins coded for a BZIP family transcription factor of unknown function (Solyc04g056510) by genes upregulated in Red Setter are assigned to function in the regulation of epidermal metabolism, especially in fruits (Solyc06g035940, ANL2; [53]). In relation to the RNA-based regulation, a nuclear pore complex protein-related gene (Solyc11g020200) is upregulated in Red Setter at 45 °C when compared to LA1994 and LA2661. In *A. thaliana*, several nuclear pore complex members are involved in temperature-regulated mRNA export but also in controlling the nuclear accumulation of temperature-response related transcription factors [54].

While the global expression pattern of stress- or redox-related genes is not different among the different genotypes, some of the genes are significantly regulated with respect to the thermotolerance profile. For example, Solyc11g011080, which is enhanced in Red Setter and Moneymaker at 25 °C codes for a putative disease resistance protein, while Solyc11g018800 which is higher expressed in Red Setter and Moneymaker at 39 °C codes for a peroxidase LePrx26 involved in pathogen response [55]. However, most of the genes of this category are found to be differentially regulated at 45 °C. For instance, a putative glutaredoxin (Solyc06g008760) is downregulated in Red Setter at 45 °C. Glutaredoxins are important for redox regulation, which is essential for thermotolerance, and it has been previously shown that overexpression of an *A. thaliana* glutaredoxin enhances both drought and HS tolerance in tomato [56,57]. In contrast, Solyc01g081250, Solyc09g005420 and Solyc11g011010 are upregulated at 45 °C in Red Setter when compared to the other genotypes. Solyc01g081250 codes for a glutathione S-transferase (HSP26A) which is involved in protection against oxidative damage e.g., [58], Solyc11g011010 codes for a putative necrosis-inducing virulence protein and Solyc09g005420 for a Major latex protein-like 28, both involved in pathogen response. Thus, these genes might be involved in the regulation of processes under distress conditions.

## 5. Conclusions

The T_50%_ value and the Hill coefficient of the growth response to different temperature treatments have been found to be a very suitable measure for classification with respect to thermosensitivity. By that, these values can serve as powerful selection parameters for future studies on understanding thermotolerance. Further, the analysis of the transcriptome at different temperatures uncovered global and genotype-specific reactions. The general HSR is comparable between thermotolerant and thermosensitive genotypes, at least at the seedling state. The system depends on stress response and energy production, the latter reflected by massive alteration of genes involved in photosynthesis or mitochondrial ATP production. The difference of the expression of hormone-related genes at 25 °C shows that the hormone status could be one characteristic defining the capacity for HSR already at the ground state. Moreover, the differential regulation of some hormone-related genes is consistent with the importance of hormone-based regulation for the fine-tuning of thermotolerance. In line, genes involved in hormone-based regulation are represented in the pool of genes with an expression profile associated with thermotolerance capacity. Interestingly, HsfA6a could represent a putative link between hormone-based gene regulation and HSR at 39 °C. Other genes are involved in RNA-based, as well as stress and redox regulation. Many of these specific genes are already differentially expressed under non-stress conditions in the tomato genotypes, and in general they are at steady state levels at all temperatures. This suggests that growth inhibition in response to HS is genetically predetermined and at least in part independent of the activation of response mechanisms. In addition, some of the genes enhanced in thermosensitive genotypes at 45 °C might be involved in distress pathways. However, we also observed a set of genes involved in genes with expression profile associated with thermotolerance capacity, including HsfA6b, should be considered for further future investigations for tomato HS resilience improvement. Several genes that we identified as putative positively related to thermotolerance in tomato seedlings have not been previously related to HS resilience, and therefore can be considered as novel targets for tomato improvement.

## Supplementary Materials

The following are available online at https://www.mdpi.com/2073-4425/11/6/655/s1, Figure S1: Comparison of the MACE and qRT-PCR results for ten selected genes. Figure S2: PC analysis of the expression profile for each genotype at different temperatures. Figure S3: The comparison of the change of transcript abundance for genes in a class in general and for genes according to the PCA-based selection. Figure S4: PC analysis of the expression profile for all genotypes at each temperature. Figure S5: Box plot representation of selected categories. Figure S6: Genes involved in hormone-based regulation. Table S1: Oligonucleotides used for qRT-PCR. Table S2: Mapping information of the MACE datasets. Table S3: Contribution of the individual PCs to the variance in the tested combinations. Table S4: The contribution of individual genes to PC1–PC5 in the global PCA. Table S5: The contribution of individual genes to PC1–PC4 for the temperature focused analysis. Table S6: The DeSeq2 analysis of the change of expression in the different genotypes. Table S7: Categories used for analysis. Table S8: Genes selected dominating the PC1 and PC2 of the temperature dependent analysis to identify species variations. Table S9: Genes involved in RNA-based regulation contributing to PC1 and PC2 while analyzing the temperature dependent profile. Table S10: Genes involved in DNA-based regulation contributing to PC1 and PC2 while analyzing the temperature dependent profile. Table S11: Genes involved in photosynthetic processes contributing to PC1 and PC2 while analyzing the temperature dependent profile. Table S12: Genes differential regulated between heat sensitive and tolerant genotypes. Table S13: Genes involved in hormone synthesis and hormone-based signaling contributing to PC1 and PC2 while analyzing the temperature dependent profile.
