# Supplementary material for: Transcriptional Basis for Differential Thermosensitivity of Seedlings of Various Tomato Genotypes
Source: Genes (Basel). 2020 Jun 16;11(6):655. doi: 10.3390/genes11060655 (PMC7349527; doi:10.3390/genes11060655)
Supplement: Supplementary file 1 [file genes-11-00655-s001.zip › Supplements/Hu 2020 FINAL MDPI Genes Supp.pdf]

Supplementary Material to:

Transcriptional basis for differential thermosensitivity of seedlings of various tomato genotypes

by Yangjie Hu, Sotirios Fragkostefanakis, Enrico Schleiff and Stefan Simm

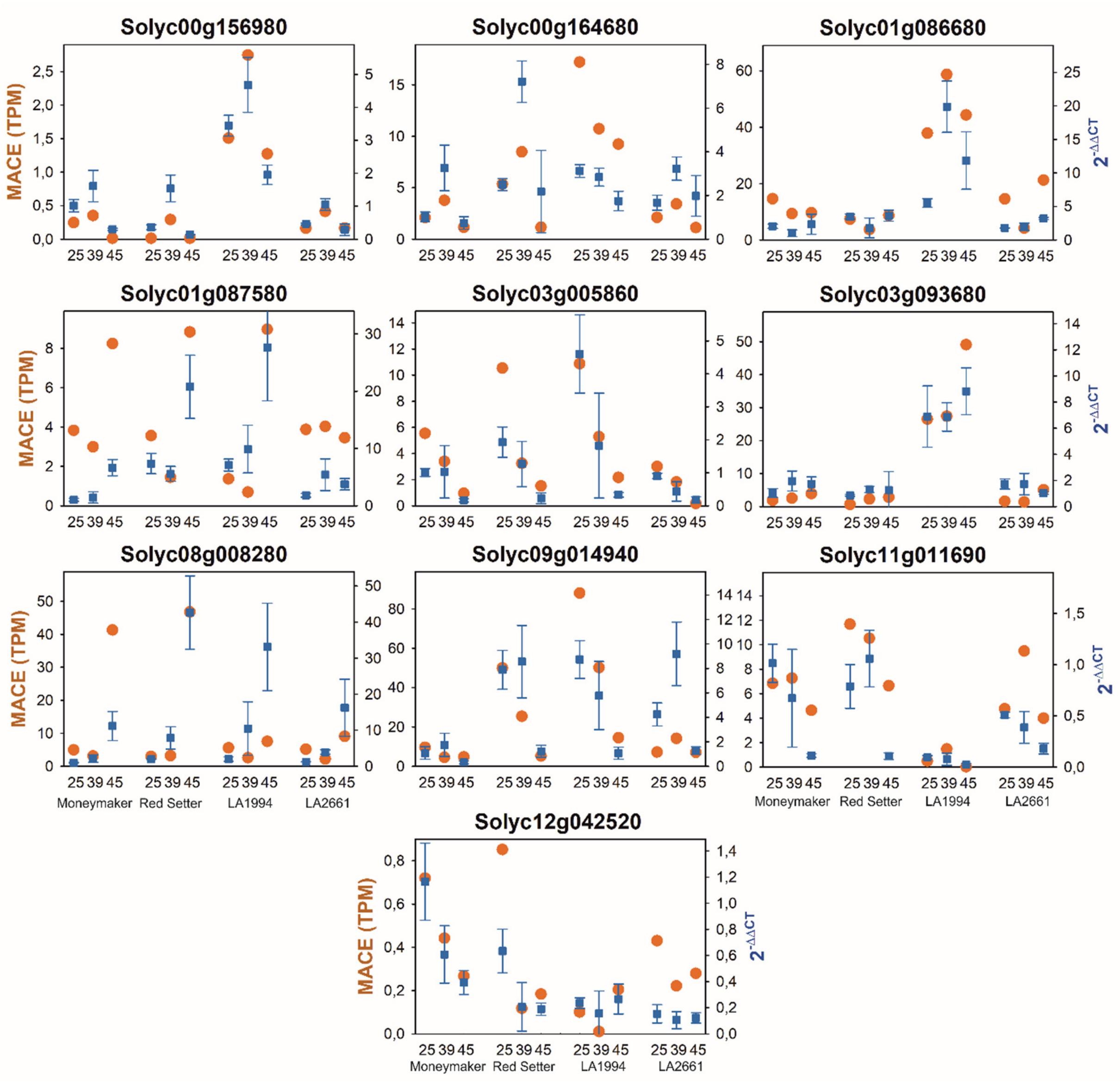

Figure S1. Comparison of the MACE and qRT-PCR results for ten selected genes. Shown is the mean TPM value for each of the conditions and genotypes (orange) determined by MACE and the transcript level via $2^{-\Delta\Delta CT}$ determined by qRT-PCR (blue) as described (methods). The standard deviation of the qRT-PCR result is shown. The values were used to calculate the Pearson correlation coefficient between the profile obtained by MACE and by qRT-PCR, which is depicted in Figure 2.

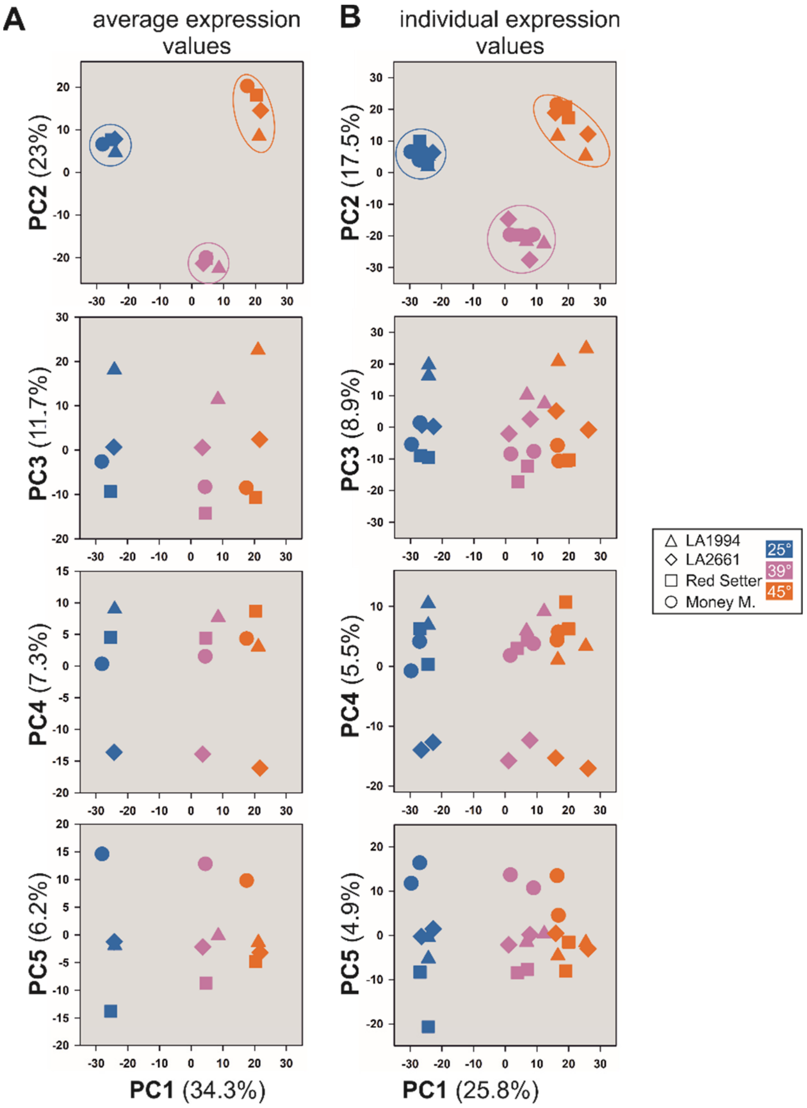

C

	PC1<br>genes / % contribution	PC2<br>genes / % contribution	OL<br>genes
Money Maker	406 / 27.8%	316 / 21.3%	105
Red Setter	433 / 29.0%	301 / 19.4%	98
LA1994	360 / 26.3%	245 / 14.1%	61
LA2661	461 / 28.2%	344 / 22.4%	57

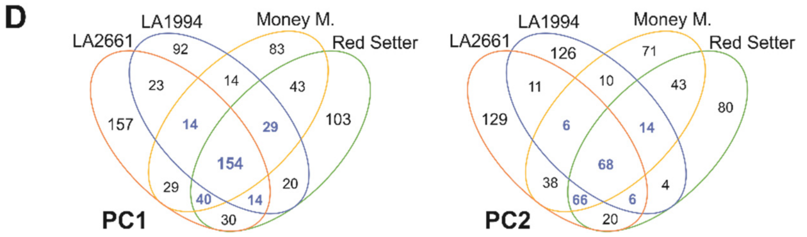

Figure S2. PC analysis of the expression profile for each genotype at different temperatures. The principal components for the mean values (A) and for the individual values (B) were calculated. The distribution of PC1 to PC5 covering 80% of the variance while analyzing the mean values is shown and the percentage of the contribution of each PC is indicated. The symbol and color legend is indicated on the right. Note, the subsequent PCs of the analysis of the individual expression values cover experimental variances. (C) The number of selected genes according to the criteria described (methods) and the total percentage of the contribution of the expression profile of all selected genes to PC1 or PC2, respectively, is shown for each cultivar. The overlap of the genes contributing to PC1 and PC2 is shown. (D) The genes contributing most to PC1 (left) or PC2 (right) according to the criteria (methods) have been selected and the overlap between different genotypes is shown. Approximately 18% (154 out of 845) of the genes contributing to PC1 are found in all genotypes and approximately 30% (251 out of 845) in at least three genotypes. In contrast, only 10% (68 out of 692) of all genes contributing to PC2 are found in all and nearly 23% (160 out of 692) in at least three genotypes.

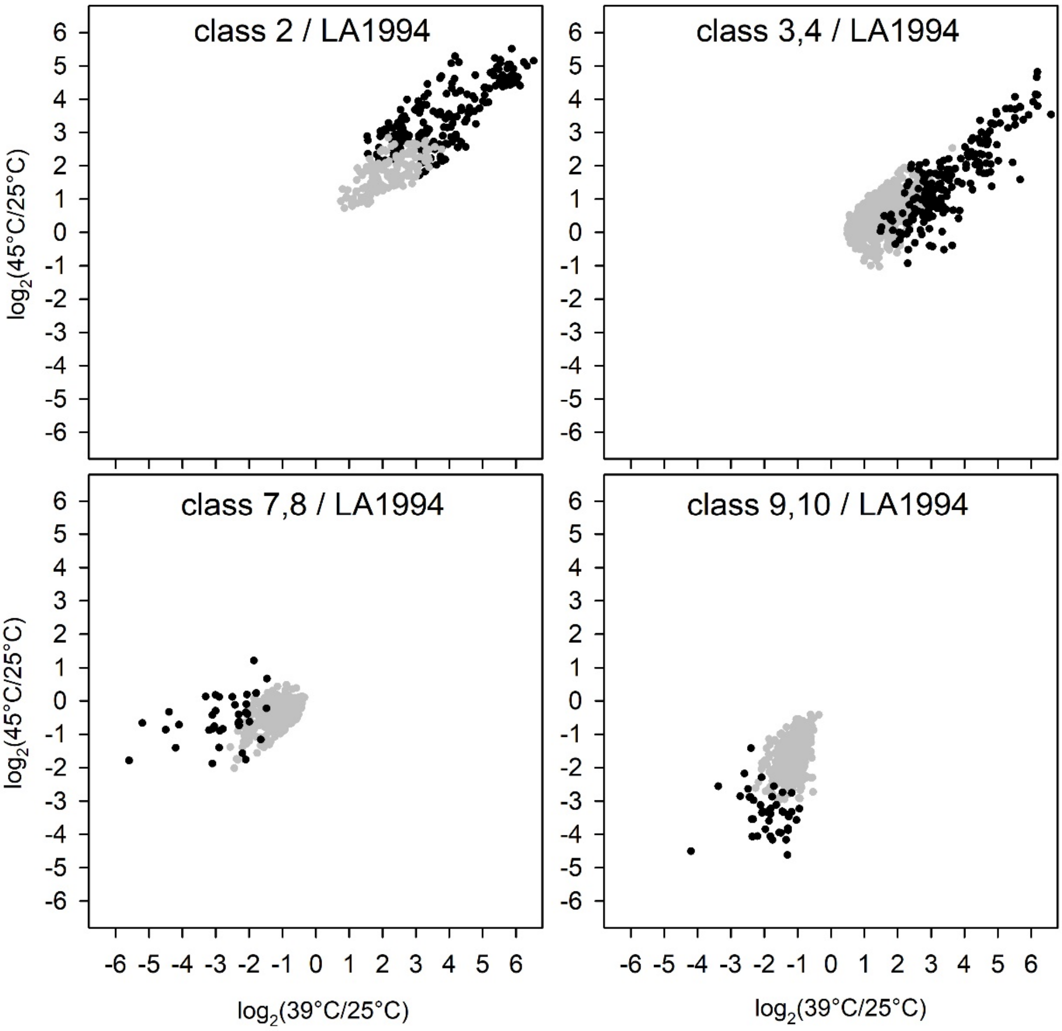

Figure S3. The comparison of the change of transcript abundance for genes in a class in general and for genes according to the PCA based selection. The change of transcription in LA1994 between 25°C and 39°C (x-axis) and 25°C and 45°C (y-axis) of genes in the indicated classes is shown. The genes selected by PCA as well are indicated in black, the other genes in the same classes in grey. This result shows that the PCA based approach selects genes that are found by differential expression via DeSeq2 but with higher log_2 foldchange values. The distribution of the genes selected by the PCA based method further shows that not a strict cut off is observed, because in contrast to the pairwise analysis by DeSeq2 the information of all temperatures are included.

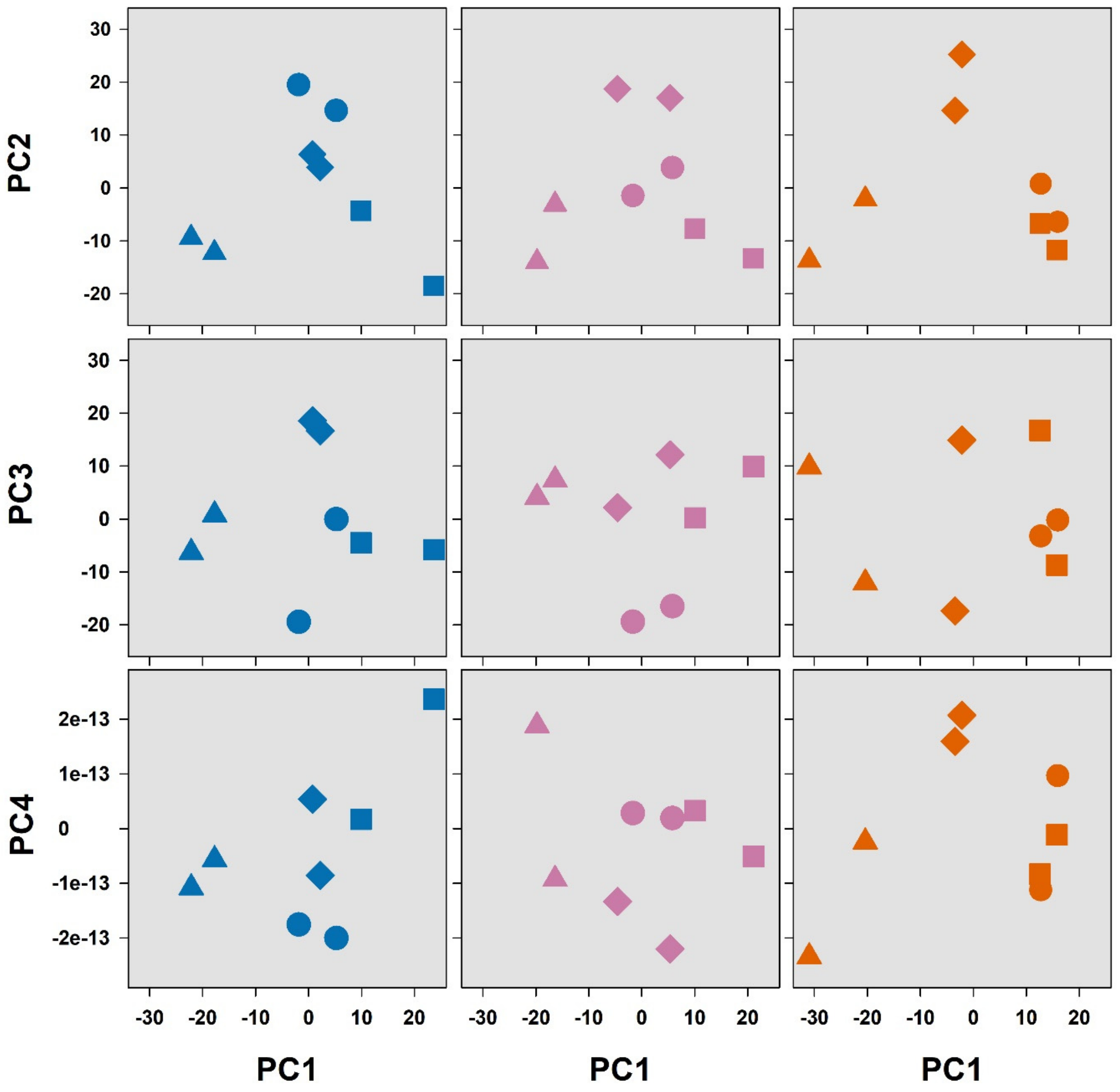

Figure S4. PC analysis of the expression profile for all genotypes at each temperature. The principal components 1 and 2 (top), 1 and 3 (middle), and 1 and 4 of the distribution of the transcript levels according to our TPM results for the individual temperatures for all genotypes was calculated for the individual datasets. The symbol and color legend is indicated in Figure 4.

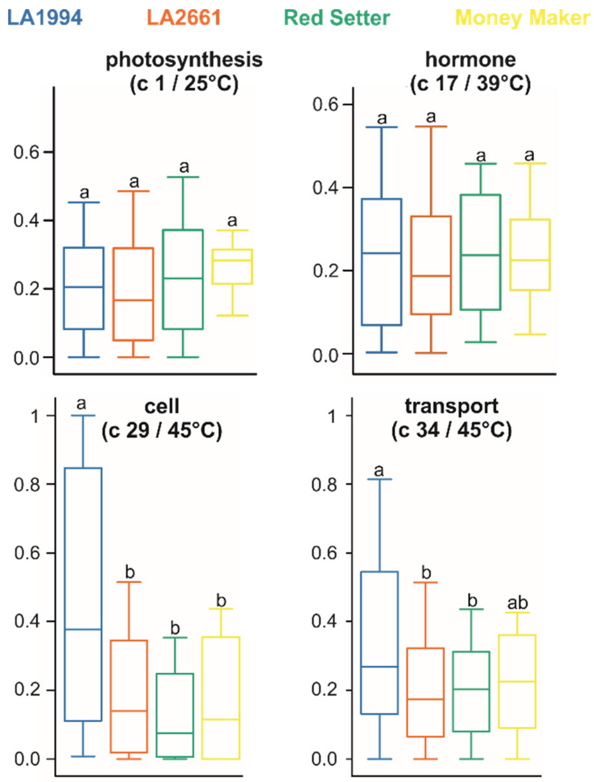

Figure S5. Box plot representation of selected categories. The box plot representation of the distribution of the normalized expression values used for the voronoid representation for the indicated class and temperature shown Figure 4 (25°C), Figure 5 (39°C) or Figure 6 (45°C) is show. Statistical analysis of the difference was analyzed by One Way ANOVA with $p<0.05$. The color code for the boxes is given on top.

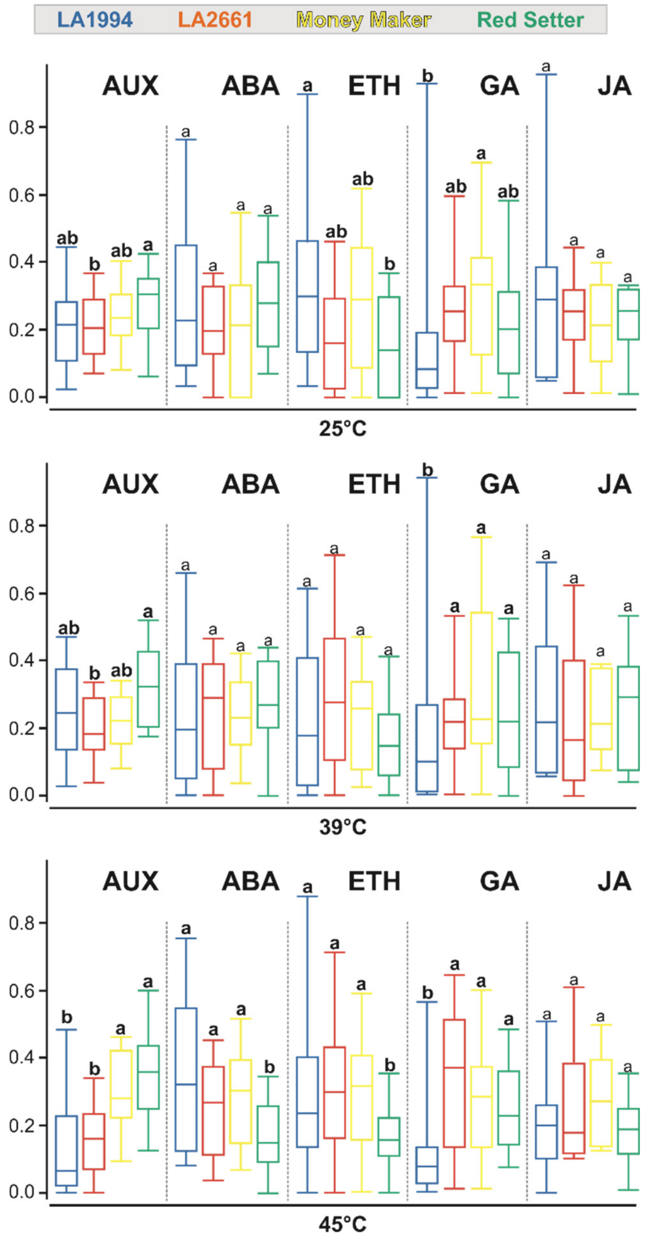

Figure S6. Genes involved in hormone based regulation. In total, 79 genes are found in the category hormons to be the most contributing to PC1 and PC2 (Figs. 5-7). An additional 22 genes most contributing to PC1 and PC2 in other categories where previously assigned as related to hormone regulation [54]. From the total of 101 genes (Table S12) the majority of genes, are assigned to abscisic acid (15 genes), ethylene (22 genes) and auxin pathways (38 genes), while fewer genes are related to jasmonic acid (10 genes), to gibberellic acid (10 genes), cytokinin (5 genes not shown) and salicylic acid (6 genes, not shown) based regulation. The box plot representation of the distribution of the normalized expression values for the genes involved in auxin or gibberellic acid based regulation used for the Voronoi representation in Figs. 5-7 is shown for 25°C (top), 39°C (middle) or 45°C (bottom). Statistical analysis for each temperature was done by One Way ANOVA as in Fig. 5C.

Table S1: List of oligonucleotides used for qRT-PCR analysis.

Table S2: Mapping information of the MACE datasets. For the three temperature regimes (column 2-4) the total number of reads and the percentage of mapped reads to the reference genome of S. lycopersicum genotype Heinz 1706 (2 biological replicates) of the four genotypes (column 1) are shown. The number of genes with assigned reads is displayed for each genotype and regime (merged replicates).

Table S3. Contribution of the individual PCs to the variance in the tested combinations. The principal components of the variation between genotypes and/or conditions were calculated using the mean of the replicates for one genotype at one condition (A and B) or the values for all individual samples (C and D). A and C contain the PCs obtained while analyzing the variance at all different temperatures and all cultivars (ALL). B and D contain the PCs calculated for the genotypes at a given temperature (right). The percentage of contribution to the description of the variance is given. The last column of B and D gives the accumulating average of the contribution of PC1, PC1 & PC2 etc.

Table S4. The contribution of individual genes to PC1 – PC5 in the global PCA. The contribution of each gene to each individual PC for each genotype was calculated as described in Materials and Methods and is given as fraction to 100%.

Table S5. The contribution of individual genes to PC1 – PC4 for the temperature focused analysis. The contribution of each gene to each individual PC for each genotype was calculated as described in Materials and Methods and is given as fraction to 100.

Table S6. The DeSeq2 analysis of the change of expression in the different genotypes. The accession number, the mean value of expression, the $\log_2$ foldchange of the transcript abundance, the lfcSE value, the stat value, the p-value and the adjusted p-value (padj) is shown for all genes where an expression was determined and where the p-value is smaller than one. The values are shown for the ratio of the values determined for 39°C and for 25°C, for 45°C and 25°C and for 45°C and 39°C.

Table S7. Categories used for analysis. The assignment of all analyzed categories to numbers is given.

Table S8. Genes selected dominating the PC1 and PC2 of the temperature dependent analysis to identify species variations. The accession number, the category and the functional description or the similarity assignment to A. thaliana proteins is given in the third column.

Table S9. Genes involved in RNA based regulation contributing to PC1 and PC2 while analyzing the temperature dependent profile. As information the accession number, the classification according to the functional category and the gene of *A. thaliana* showing the highest similarity. Subsequently, the average value of the expression as $\log_{10}$ for each temperature and each genotype is listed.

Table S10. Genes involved in DNA based regulation contributing to PC1 and PC2 while analyzing the temperature dependent profile. As information the accession number, the classification according to the functional category, the gene of *A. thaliana* showing the highest similarity, and the assignment of a putative function is given. Subsequently, the average value of the expression as $\log_{10}$ for each temperature and each genotype is listed.

Table S11. Genes involved in photosynthetic processes contributing to PC1 and PC2 while analyzing the temperature dependent profile. As information the accession number, the classification according to the functional category, the gene *of A. thaliana* showing the highest similarity in *A. thaliana*, the gene name either of the tomato gene or of the similar *A. thaliana* gene and the assignment to the different photosynthetic complexes / pathways is given. Subsequently, the average value of the expression as $\log_{10}$ for each temperature and each genotype is listed.

Table S12. Genes differential regulated between heat sensitive and tolerant genotypes. All selected according to the method section 2.8 for the indicated temperature is shown. Indicated is whether the genes is selected as differentially regulated at 25°C (column 1), 39°C (column 2) or 45°C column 3). The column 4 shows the accession number. Column 5-12 show the $\log_2$ value and the p-value for the indicated ration (MM: Moneymaker; RS: Red Setter). Column 13 indicates the class the

genes is assigned to, column 14 the abbreviation of the genes, column 15 the functional assignment and column 16 the category.

Table S13. Genes involved in hormone synthesis and hormone based signaling contributing to PC1 and PC2 while analyzing the temperature dependent profile. As information the hormone to which the gene is functionally assigned, the accession number and the number of the functional category. Subsequently, the average value of the expression as $\log_{10}$ for each temperature and each genotype is listed.

Table S1. List of oligonucleotides used for qRT-PCR analysis.

Gene name	Acc. number	Forward primer (5'->3')	Reverse primer (5'->3')
EF1a	Solyc06g005060	GGAACTTGAGAAGGAGCCTAAG	CAACACCCACAGCAACAGTTT
Choline dehydrogenase	Solyc00g156980	AATGGCTGTGGTTGGTCCTCTC	CCTTCACCTCCGCTTCCTTGAATG
Gibberellin-regulated family protein	Solyc12g042520	AGCAGGACGACAAGACAGATGC	AGCAAGGGCACTCATCTTTATGCC
Wound-induced protein 1	Solyc09g014940	ACTGAGTTGAGGCCTATGGGTTGG	TTGCTAGCATAAGGCCGGGAAG
Unknown Protein	Solyc01g087580	GCTGGAATTAGAATTGCCGCTAGG	GCGATCGTCATCGGAGTATTGC
Auxin-responsive protein	Solyc11g011690	CAGCTGATCAAGTTCGCTTTGGC	AGTCCATGAAGGCTGAATCACAGG
glutathione S-transferase T1	Solyc01g086680	TGGAGGAGACAGCATTGGATTCG	TGAATTGCTCCAAACCAGAGAGC
WRKY transcription factor 53	Solyc08g008280	TCTGCACCACAACCAACATCGC	TGCTGCCCTGTTGGATAAACGG
Lipid A export ATP-binding/permease protein msbA	Solyc03g005860	ACGACCCTGCAAACTCTTGTGG	ATCTCCCGCCAAATTCCGGATAG
Mago nashi-like protein 2	Solyc03g093680	TGAGTTTAGGCCTGATGGCAAGC	TCTCGCTATCAGCAACAATGCG
ABC transporter G family member 11	Solyc00g164680	TGATCTTGGCTACTACTGGATGCG	AGCCACCATTAGACCTCTTTCCTC

Table S2: Mapping information of the MACE datasets
For the three temperature regimes (column 2-4) the total number of reads and the percentage of mapped reads to the reference genome of *S. lycopersicum* genotype Heinz 1706 (2 biological replicates) of the four genotypes (column 1) are shown. The number of genes with assigned reads is displayed for each genotype and regime (merged replicates).

	**Temperature**	**25°C**		**39°C**		**45°C**	
	**Cultivar**	Rep A	Rep B	Rep A	Rep B	Rep A	Rep B
**# Mio reads**	Moneymaker	5.56	9.22	9.17	7.50	7.65	5.50
	Red Setter	8.88	4.90	9.87	6.00	5.74	6.24
	LA1994	8.30	8.52	9.66	8.47	8.90	5.78
	LA2661	10.85	7.26	10.73	8.91	7.44	6.27
**% mapped**	Moneymaker	86.5	88.5	87.5	87.9	83.4	85.3
	Red Setter	86.6	84.9	86.4	88.0	80.4	84.3
	LA1994	85.9	86.8	86.3	87.5	81.9	84.6
	LA2661	87.0	86.4	86.8	87.2	82.4	84.2
**# genes**	Moneymaker	21,647		21,665		21,854	
	Red Setter	21,203		21,609		21,500	
	LA1994	21,909		21,900		22,029	
	LA2661	21,662		22,191		21,848	
